# Positron emission tomography of tumour [^18^F]fluoroestradiol uptake in patients with acquired hormone-resistant metastatic breast cancer prior to oestradiol therapy

**DOI:** 10.1007/s00259-015-3107-5

**Published:** 2015-06-20

**Authors:** Michel van Kruchten, Andor W. J. M. Glaudemans, Erik F. J. de Vries, Carolien P. Schröder, Elisabeth G. E. de Vries, Geke A. P. Hospers

**Affiliations:** Department of Medical Oncology, University Medical Centre Groningen, University of Groningen, PO Box 30.001, 9700 RB Groningen, The Netherlands; Department of Nuclear Medicine and Molecular Imaging, University Medical Centre Groningen, University of Groningen, Groningen, The Netherlands

**Keywords:** Oestrogen receptor, Imaging, Breast cancer, Oestradiol therapy, ^18^F-FES-PET

## Abstract

**Purpose:**

Whereas anti-oestrogen therapy is widely applied to treat oestrogen receptor (ER) positive breast cancer, paradoxically, oestrogens can also induce tumour regression. Up-regulation of ER expression is a marker for oestrogen hypersensitivity. We, therefore, performed an exploratory study to evaluate positron emission tomography (PET) with the tracer 16α-[^18^F]fluoro-17β-oestradiol (^18^F-FES) as potential marker to select breast cancer patients for oestradiol therapy.

**Methods:**

Eligible patients had acquired endocrine-resistant metastatic breast cancer that progressed after ≥2 lines of endocrine therapy. All patients had prior ER-positive histology. Treatment consisted of oestradiol 2 mg, three times daily, orally. Patients underwent ^18^F-FES-PET/CT imaging at baseline. Tumour ^18^F-FES-uptake was quantified for a maximum of 20 lesions and expressed as maximum standardised uptake value (SUV_max_). CT-scan was repeated every 3 months to evaluate treatment response. Clinical benefit was defined as time to radiologic or clinical progression ≥24 weeks.

**Results:**

^18^F-FES uptake, quantified for 255 lesions in 19 patients, varied greatly between lesions (median 2.8; range 0.6–24.3) and between patients (median 2.5; range 1.1–15.5). Seven (37 %) patients experienced clinical benefit of oestrogen therapy, eight progressed (PD), and four were non-evaluable due to side effects. The positive and negative predictive value (PPV/NPV) of ^18^F-FES-PET for response to treatment were 60 % (95 % CI: 31–83 %) and 80 % (95 % CI: 38–96 %), respectively, using SUV_max_ >1.5.

**Conclusion:**

^18^F-FES-PET may aid identification of patients with acquired antihormone resistant breast cancer that are unlikely to benefit from oestradiol therapy.

## Introduction

Until the introduction of tamoxifen, additive oestrogens such as the synthetic diethylstilbestrol (DES) were considered the hormone treatment of choice in postmenopausal women. In a randomised study in 143 postmenopausal patients with metastatic breast cancer, first-line endocrine therapy with DES was equally effective as tamoxifen with a response rate of 41 % vs. 33 %. Yet, tamoxifen became the preferred agent because it showed fewer side effects [1]. An emerging number of anti-oestrogen therapies have become available since. Recently, however, additive oestrogen therapy has regained interest by showing efficacy in ~35 % of patients that are extensively pre-treated with anti-oestrogens [2]. Interestingly, an update of the randomised study showed a superior 5-year survival for DES compared to tamoxifen (35 % vs. 16 %) after 14 years of follow-up [3]. Moreover, in a recent study a lower dose of only 6 mg oestradiol rendered similar clinical benefit rates as 30 mg oestradiol with fewer side effects. Finally, clinical results suggest that oestrogens can restore the sensitivity to anti-oestrogens [4]. As the majority of patients will not benefit from additive oestrogen therapy, a biomarker for patient selection would be helpful.

In preclinical studies, long-term oestrogen deprivation triggered hypersensitivity to oestrogens, which is accompanied by a five- to 10-fold increase in ER expression [5, 6]. Thus, patients that have been treated with anti-oestrogens for a long time may likewise have become hypersensitive to oestrogens. If so, patients that are most likely to benefit from oestradiol therapy could potentially be identified by high tumour ER expression. Positron emission tomography (PET) with 16α-[^18^F]fluoro-17β-oestradiol (^18^F-FES) can visualise and quantify ER expression in breast cancer lesions [7]. The aim of this exploratory study was, therefore, to evaluate ^18^F-FES-PET as a potential marker to select breast cancer patients for oestradiol therapy.

In the setting of ER-positive metastatic breast cancer, response assessment is notoriously difficult due to the high incidence of bone metastases. Bone is the most common site affected in breast cancer [8]. However, bone metastases are regarded non-measurable by the response evaluation criteria in solid tumours (RECIST) [9]. This underlines the need for objective measures to predict and evaluate response, for example by molecular imaging techniques. Recent studies have shown that ^18^F-FES-PET can predict response to various forms of anti-oestrogen therapy [10–13]. Its value as a biomarker for additive oestrogen therapy is, however, unknown.

In addition to molecular imaging techniques, also serum markers may be valuable to assess response in patients with bone-dominant disease in which response assessment is difficult. The ASCO recommendations for the use of tumour markers in breast cancer indicate that CA 15.3 and CEA can be considered to monitor treatment effects [14]. Also, bone turnover markers such as procollagen type I amino-terminal propeptide (PINP), carboxyl-terminal telopeptide of type I collagen (CTx), and bone alkaline phosphatase (BALP) are reported to correlate with the number and size of bone metastases in breast and prostate cancer [15–17]. Therefore, in addition to ^18^F-FES-PET, we also evaluated whether tumour markers, and bone turnover markers can aid response prediction.

## Material and methods

### Patients

The trial was conducted in accordance with the principles of Good Clinical Practice and the Declaration of Helsinki. The protocol was approved by the local medical ethics committee and registered in the ClinicalTrials.gov database (NCT01088477). All patients provided written informed consent.

Eligible patients had acquired endocrine-resistant advanced breast cancer showing progression after ≥2 lines of endocrine therapy. All patients had earlier ER-positive immunohistochemical tumour staining, and were required to have responded to at least one prior line of anti-hormone therapy (objective response or stable disease ≥6 months). Other eligibility criteria were ECOG performance ≤2 and life expectancy ≥3 months. Exclusion criteria were the presence of symptomatic central nervous system lesions, a history of thrombosis, diabetes mellitus, uncontrolled hypercalcaemia, treatment with investigational drugs within 30 days before the start of study, dyspnoea at rest due to any cause, and class III or IV congestive heart failure according to the New York Heart Association. Patients were required to withdraw drugs known to bind ER for at least 5 weeks prior to baseline imaging [18].

### Oestradiol treatment

Patients were treated with oestradiol three times daily 2 mg orally [4]. Therapy was initiated within 4 days after ^18^F-FES-PET/CT. In case of toxicity oestradiol dosing was reduced to twice daily 2 mg, or shortly interrupted with re-introduction at a lower dose when the symptoms had resolved. Therapy was continued until progressive disease (PD) by radiologic or clinical assessment, withdrawal of consent, or severe toxicity. Toxicity was documented according to the Common Terminology Criteria of Adverse Events v3.0.

### Assessment of treatment response

Baseline measurements included documentation of all symptoms, performance status, physical examination, laboratory tests (including blood counts, kidney function, and liver enzymes), and a diagnostic CT-scan. Clinical follow-up with documentation of symptoms, performance status, physical examination, and laboratory tests were done monthly. A diagnostic CT-scan was performed every 3 months until progression. For patients with measurable disease, response was defined according to RECIST v1.1 [9]. Patients with only non-measurable lesions were considered to have PD when there was unequivocal progression of existing lesions or when new lesions were detected at follow-up. In the absence of radiological PD, patients could develop clinical PD, defined as an overall level of substantial worsening such that the overall tumour burden or complaints increased sufficiently to merit discontinuation of therapy [9]. In reference to other studies, patients with time-to-progression ≥24 weeks were considered to have obtained clinical benefit from oestradiol therapy [19].

### Study measurements

**Fig. 1:**
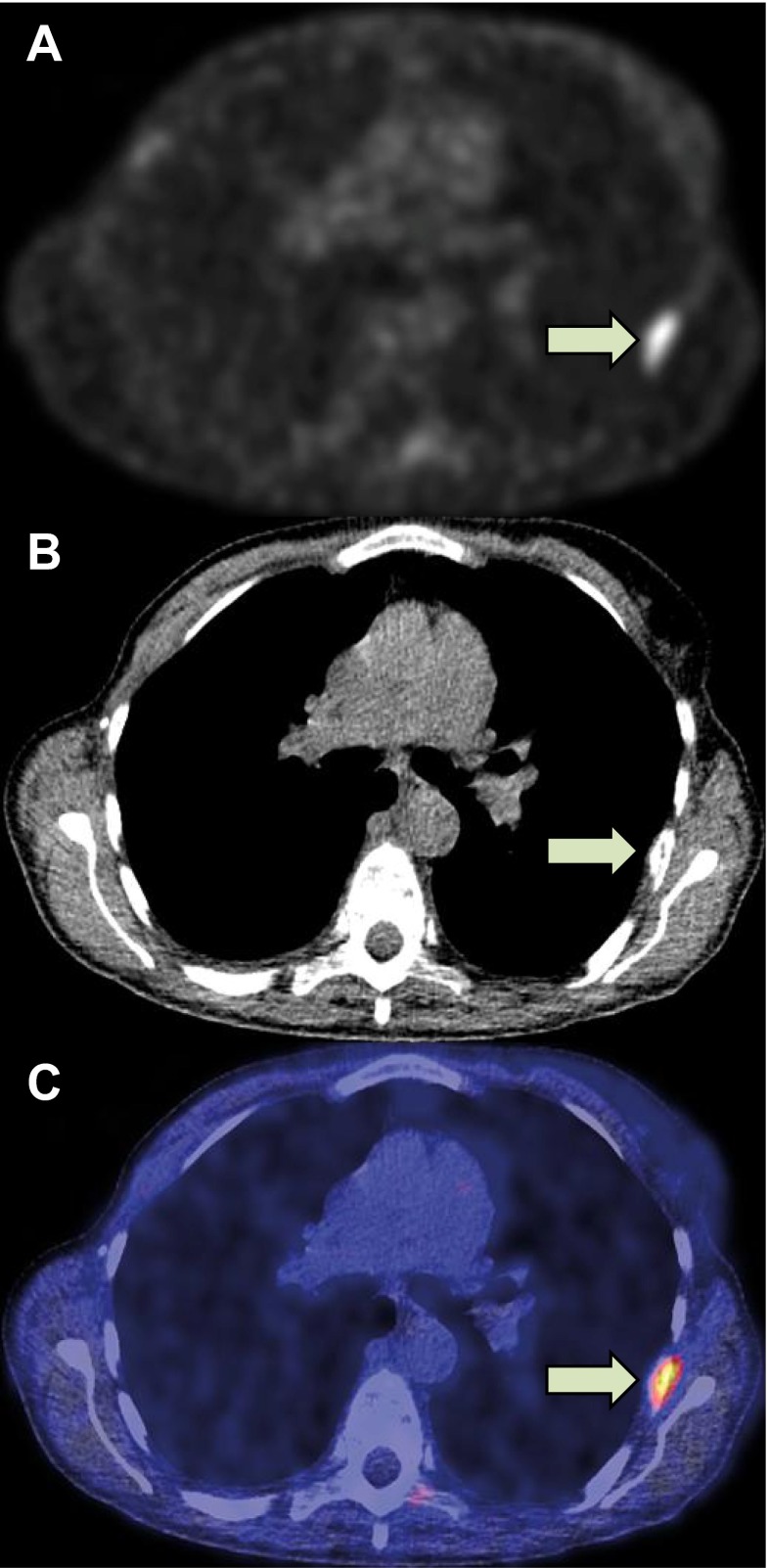
^18^F-FES-PET (**a**), CT (**b**) and ^18^F-FES-PET/CT (**c**) of a patient with bone metastases. Indicated is a rib metastasis (*arrow*) with SUV_max_ 3.3

^18^F-FES was produced and administered to the patient as described earlier [18, 20]. On average 3.4 ± 1.5 GBq ^18^F-FES was obtained with 100 % radiochemical purity and a 325 ± 274 GBq/μmol specific activity. Patients received approximately 200 MBq ^18^F-FES intravenously. ^18^F-FES-PET/CT to evaluate tumour ER-expression was performed at baseline on a hybrid PET/CT camera with a 64-slice CT and high definition and time-of-flight PET (Siemens Medical Systems). Low-dose CT-scan was used for attenuation correction in all patients. Patients were scanned from skull to mid-thigh, 3 min per bed position (usually 7–8 bed positions per patient). In all patients, baseline ^18^F-FES-PET was combined with a contrast-enhanced diagnostic CT scan. For representative ^18^F-FES-PET, CT and ^18^F-FES-PET/CT images see Fig. 1.

Tumour ^18^F-FES uptake was quantified by a nuclear medicine physician according to the guidelines of the European Association of Nuclear Medicine (EANM) for an arbitrary maximum of 20 randomly chosen lesions per patient [21]. Whole-body CT-scan was evaluated by a radiologist and used to allocate tumour lesions and identify possible ^18^F-FES-negative lesions. Lesion ^18^F-FES-uptake was expressed as maximum standardised uptake value (SUV_max_). To calculate the predictive value of ^18^F-FES-PET for treatment response in individual patients, the median SUV_max_ of all lesions quantified was used. Quantification of tumour ^18^F-FES-uptake was performed while blinded for treatment outcome. Patients and treating physician were held blinded for ^18^F-FES-PET results.

Tumour markers (CA-15.3, CEA) and bone turnover markers (PINP, CTx, and BALP) were also determined at baseline and repeated every 3 months or at the time of progression. Patients were considered evaluable for tumour marker response if one or both tumour markers were increased at baseline (CA15.3 > 33 kU/L, CEA >5 μg/L). A 10 % increase in tumour marker was defined as biochemical progression. Patients were considered evaluable for bone turnover markers when they had evidence of bone metastases on imaging. Serum PINP >95 ng/mL was considered the threshold for increased bone turnover based on literature [17]. For CTx and BALP, the optimum thresholds were determined by receiver operating characteristic (ROC) analysis.

### Statistical analysis

The expected study time frame was 3 years for inclusion of 50 patients to evaluate the positive predictive value (PPV) and negative predictive value (NPV) of ^18^F-FES-PET/CT for response to oestradiol therapy by ROC analysis. After 3 years and 21 patients included, the study was terminated. We here report the PPV and NPV for ^18^F-FES-PET/CT, which was the predefined primary end point of the study. PPV and NPV were calculated using a ROC analysis for the median tumour SUV_max_ in patients. The association between biochemical tumour markers and bone turnover markers and benefit from oestradiol was calculated by a Mann–Whitney *U* test. Analyses were performed in SPSS Statistics version 20.0.

## Results

### Patient characteristics

**Fig. 2:**
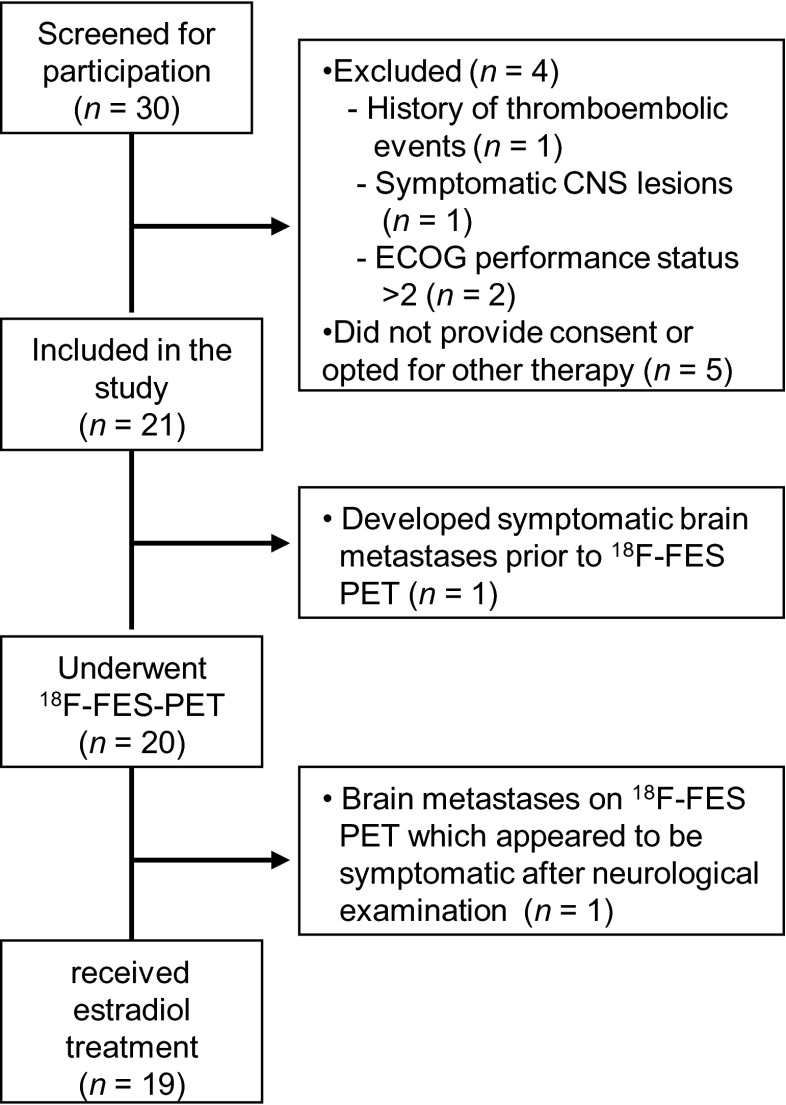
CONSORT diagram. *CNS* Central nervous system, *ECOG* Eastern Cooperative Oncology Group

Between May 2010 and May 2013, 30 patients were screened for participation in the trial, out of whom 21 were included and 19 started oestradiol therapy, one man and 18 women. Mean age was 57 years (range 36–76). Seventeen patients had bone metastases; 14 patients also had visceral or nodal metastases. Two patients had only visceral lesions. All patients had postmenopausal status, which was in two patients achieved by the use of LHRH agonists, while others were truly postmenopausal. Tumour histology was positive for ER in all patients, 12 (63 %) were also PR positive, and none were HER2 positive. All patients were heavily pre-treated; 11 patients had already received 3–4 lines of systemic therapy, and seven patients ≥5 lines. Patient characteristics are summarised in Table 1. For an overview of screening, inclusion, and exclusion see the CONSORT diagram (Fig. 2).

**Table 1:** Patient characteristics

Baseline characteristics	All patients (*n* = 19)
Male : female	1 : 18
Age, mean years (range)	57 (36–76)
Site of metastases, *n*	
Bone + visceral	14
Bone-only	3
Visceral-only	2
Measurable lesions, *n*	
Measurable visceral	12
Non-measurable visceral	4
Bone-only	3
Tumour receptor expression	
ER positive	19
Progesterone receptor positive	12
HER2 positive	0
Prior systemic therapies, *n*	
< 3 lines	1
3 or 4 lines	11
≥ 5 lines	7
Menopausal status, *n*	
Postmenopausal	13
Following ovariectomy	3
Goserelin treatment	2

### Tumour response

Twelve patients had measurable lesions on baseline CT according to RECIST, four patients had non-measurable visceral lesions, and three patients had only bone metastases. Four of 19 (21 %) discontinued oestradiol because of side effects and were, therefore, not evaluable for treatment response.

Seven of the remaining 15 patients experienced clinical benefit from oestradiol therapy as indicated by stable disease ≥24 weeks. Four had radiological measurable stable disease, and three patients had no new lesions detected on radiological examination, no progression of non-measurable lesions, improvement or stabilization of symptoms, and no evidence of biochemical progression ≥24 weeks. They eventually experienced PD according to RECIST criteria at 26, 28, and 48 weeks, respectively.

Finally, eight patients had PD; in five there was radiologic PD and in three patients there was substantial clinical deterioration, meriting discontinuation of therapy. One had laboratory signs of bone marrow invasion, confirmed with a biopsy; one had rising liver function test values, a threefold increase in tumour marker CA15.3, and clinical deterioration; and one patient had deterioration of pain symptoms from bone lesions, rising alkaline phosphatase, and worsening of performance score. Overall clinical benefit rate was 37 % in all treated patients (intention-to-treat; *n* = 19 patients) and 47 % in all patients available for response assessment (*n* = 15 patients). Mean progression-free-survival was 4.7 months (range 0.4–15.3 months). Oestradiol therapy induced an increase in serum oestradiol levels from 89 ± 15 pmol/L at baseline to 1241 ± 225 pmol/L after 1 month of therapy. The increase in oestradiol levels was equal among responders and non-responders.

### Toxicity

In four patients (21 %), oestradiol therapy was terminated prematurely due to adverse events. These side effects were progressive thrombocytopenia (*n* = 1), transient ischemic accident with atrial fibrillation (*n* = 1), mood disorders (*n* = 1), and signs of congestive heart failure (*n* = 1). Other grade 3 serious adverse events requiring hospital admission were tumour flare (*n* = 1), hypercalcaemia (*n* = 2), pneumonia (*n* = 1), and atrial fibrillation (*n* = 1). The patient who experienced a tumour flare had rapid increase of pain symptoms at known metastatic sites, starting as early as the day after initiation of oestrogen therapy. Laboratory results were suggestive of tumour flare with increased lactate dehydrogenase and other liver enzymes. Symptoms and laboratory findings resolved after oestradiol discontinuation. Interestingly, this patient with grade 3 clinical flare reaction showed highest tumour uptake of ^18^F-FES (median SUV_max_ 15.5, maximum SUV_max_ 24.3). Common, but manageable grade 1–2 adverse events were tumour flare, fatigue, nausea, and vaginal bleeding.

### Predictive value of ^18^F-FES-PET for response to oestradiol therapy

**Fig. 3:**
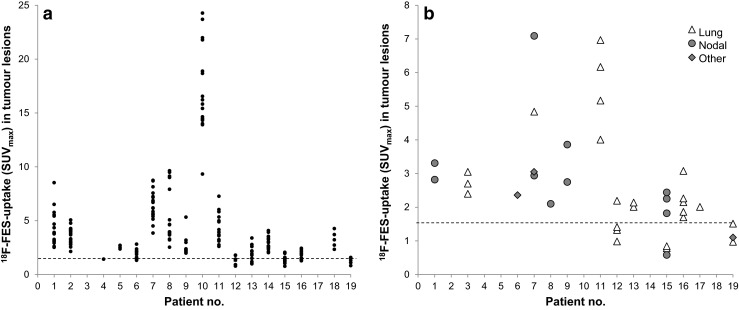
^18^F-FES-uptake (SUV_max_) in bone lesions (**a**) and non-bone lesions (**b**) in all individual patients. The *dashed line* indicates the 1.5 (SUV_max_) threshold

**Fig. 4:**
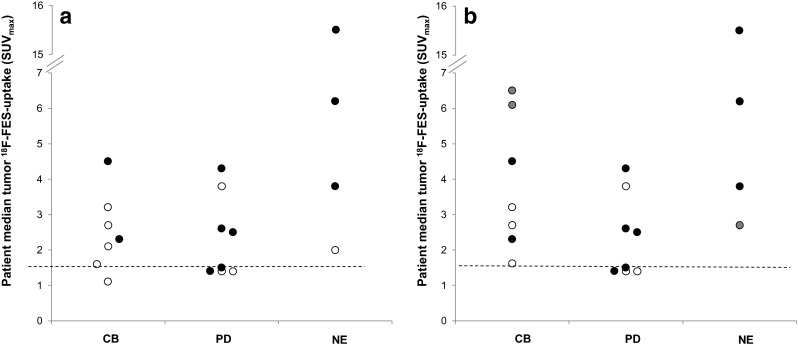
**a** Association between ^18^F-FES-uptake and treatment outcome. Patients with clinical benefit (CB), progressive disease (PD), and non-evaluable (NE) patients are indicated. The *dashed line* indicates the 1.5 (SUV_max_) threshold. Patients indicated in white had recently been treated with ER-antagonists. **b** In three patients, indicated in *gray*, a previous ^18^F-FES-PET scan was available. Using this scan resulted in an improvement of the PPV and NPV

^18^F-FES uptake in tumour lesions was quantified for a total of 255 lesions (214 bone, 24 lung, 12 lymph nodes, one breast, one soft-tissue, and one brain lesion) out of which 42 (16 %) were ^18^F-FES-negative (SUV_max_ <1.5). Twelve out of 19 patients (63 %) had only ^18^F-FES-positive lesions, six (32 %) had both FES-positive and FES-negative lesions, and one had only FES-negative lesions. Absolute ^18^F-FES-uptake (SUV_max_) varied widely between lesions (median 2.8; range 0.6–24.3) and patients (median 2.5; range 1.1–15.5), as is depicted in Fig. 3a and b. ROC analysis indicated that the most optimum threshold to differentiate between patients with clinical benefit and patients with PD was a median SUV_max_ of >1.5. For patients with response assessment available (*n* = 15 patients), this threshold produced a PPV of 60 % (95 % CI: 31–83 %) and an NPV of 80 % (95 % CI: 38–96 %) (Fig. 4a), with an area under curve of 0.62.

Nine patients terminated treatment with ER-antagonists 5 weeks before initiating oestrogen therapy. Three of these patients had an earlier ^18^F-FES-PET obtained in another study (NCT01377324). These patients had much lower ^18^F-FES-uptake than on the earlier scans and several lesions could no longer be observed. For example, one patient had, on earlier ^18^F-FES-PET, a median tumour^18^F-FES-uptake of 6.5, while in the current study, SUV_max_ was only 1.1. This patient benefited from oestradiol despite the relatively low tumour ^18^F-FES-uptake. The remaining six patients had no earlier scans available, but also had relatively low tumour ^18^F-FES-uptake compared to patients without recent use of drugs that can bind ER. Thus, ER-antagonists may reduce tumour ^18^F-FES-uptake beyond the currently used 5-week drug withdrawal period [18]. In an explorative analysis, using the results of the previous ^18^F-FES-PET scans instead of the current PET scans, the PPV and NPV increased to 64 and 100 %, respectively (Fig. 4b).

### Tumour markers and bone turnover markers

Seventeen patients had increased tumour markers. A tumour marker response was seen in seven patients. The PPV and NPV for tumour marker response were 67 and 71 %, respectively.

Bone turnover markers were explored as potential effect sensors in patients with bone metastases. Mean levels were 175 ng/mL for PINP (range 17–613 ng/mL), 90 U/L for BALP (range 27–298 U/L), and 467 pg/mL for sCTx (range 26–1369 pg/mL). Change in bone turnover markers was not associated with treatment response. Baseline PINP levels were 85 vs. 234 ng/mL (*P* = 0.032), and sCTx levels 195 pg/mL vs. 623 pg/mL (*P* = 0.032) in patients with clinical benefit and PD, respectively. The PPV of baseline PINP was 100 % (five out of five patients with PINP ≤95 ng/mL responded) and the NPV 88 % (seven out of eight patients with PINP >95 ng/mL progressed) [17]. All four patients with sCTx levels <200 pg/mL responded, and seven of nine patients with sCTX >200 pg/mL progressed. BALP levels were non-informative in this exploratory study. The PPV and NPV for ^18^F-FES-PET/CT, tumour markers, and the bone turnover marker PINP are shown in Table 2.

**Table 2:** Association between ^18^ F-FES-PET, tumour markers, bone marker, and treatment outcome

Marker	Result	*n*	Response			Predictive value	
			CB	PD	NE	PPV	NPV
^18^F-FES-PET	SUV ≥1.5	10	6	4	(4)	60 %	
	SUV <1.5	5	1	4	(0)		80 %
Tumour marker	R	6	4	2	(1)	67 %	
	NR	7	2	5	(3)		71 %
	NE	2	1	1	(0)		
Bone marker	<95 pg/mL	5	5	0	(1)	100 %	
PINP	≥95 pg/mL	8	1	7	(3)		88 %
	NE	2	1	1	(0)		

*CB* clinical benefit, *PD* progressive disease, *NE* non-evaluable, *PPV* positive predictive value, *NPV* negative predictive value, *R* response, *NR* non-response

## Discussion

This is the first exploratory study evaluating ^18^F-FES-PET/CT as predictive marker for oestradiol therapy in patients with metastatic endocrine resistant breast cancer. While the mechanism of anti-oestrogen therapy is well known, this is not the case for the addition of oestrogens. Based on preclinical data, we hypothesised that very high ^18^F-FES uptake would predict response to oestradiol therapy. The value of ^18^F-FES-PET, however, turned out to be especially its ability to identify patients that are unlikely to benefit from oestradiol therapy as a result of low or absent ^18^F-FES uptake in metastases.

There are currently no good upfront predictive biomarkers to select patients for oestradiol therapy. Assessing ER status by a biopsy is the current gold standard, but is sometimes unreliable due to heterogeneous ER expression within and among lesions, and detection of non-functional ER. 2′-[^18^F]fluoro-2′-deoxyglucose (^18^F-FDG) PET imaging has been tested to predict response to oestradiol therapy. A study randomised 66 patients to 6 or 30 mg oestradiol daily, 43 patients underwent ^18^F-FDG-PET imaging before and 24 h after the initiation of oestrogen therapy [4]. A metabolic flare reaction upon oestradiol therapy, predefined as a ≥12 % increase in tumour ^18^F-FDG-uptake, had a PPV of 80 % (12 of 15 patients), and an NPV of 87 % (27/31 patients) for response to oestradiol therapy. Metabolic flare on ^18^F-FDG-PET in 51 patients subsequently treated with an aromatase inhibitor or fulvestrant had an even higher PPV and NPV of 100 and 94 % [12]. ^18^F-FES-PET was evaluated earlier in three studies as a predictive biomarker before the initiation of aromatase inhibitors, tamoxifen, or fulvestrant. In these studies, the PPV of ^18^F-FES-PET ranged between 32 and 79 % and the NPV between 82 and 100 % [10–12], which is comparable with our findings. It is hypothesised that a negative ^18^F-FES-PET can identify tumours that have lost ER-expression during the course of disease [7, 10–12]. Recently, *ESR1* (ER) gene mutations have been described, some of which strongly reduce ligand binding affinity and induce endocrine resistance [22]. These phenomena might explain the good NPV of low tumour uptake of ^18^F-FES.

Our study has some limitations. First, the number of patients included was lower than expected. A possible explanation is that, despite the fact that previous studies have shown the safety and benefits of oestradiol therapy, physicians may be reluctant to refer patients for oestrogen therapy given that anti-oestrogen therapy is the key method to treat patients with ER-positive disease. Secondly, given the low number of measurable lesions in this population, our study was not powered to evaluate the correlation between ^18^F-FES uptake and response in individual lesions. Also, we used CT to identify ^18^F-FES-negative lesions. It is possible that ^18^F-FDG-PET together with ^18^F-FES-PET increases the sensitivity for ^18^F-FES-negative lesions, since bone lesions are especially difficult to characterise on CT. Finally, when evaluating the predictive value of ^18^F-FES-PET, it is important to take concomitant and recent therapies into account. We observed low ^18^F-FES-uptake in several previously ^18^ F-FES-avid lesions in patients that had used ER-antagonists up to 5 weeks before ^18^F-FES-PET. It is possible that the long half-lives (*t½*) of fulvestrant (*t½* = 40 days) and of tamoxifen and metabolites (*t½* = 9 and 13 days, respectively) are responsible for the ^18^F-FES-negative results [23–25]. However, in the absence of biopsies, we are unable to dissect fully whether the observed effects can be attributed to altered ER-expression or to spill-over effects of recent therapies.

In preclinical studies, long-term oestrogen-deprived ER-positive breast cancer cells are used to study oestrogen-induced apoptosis. After several months of culturing in oestrogen-deprived conditions, breast cancer cells adapt to the low levels of oestrogens by increasing ER expression [26, 27]. Paradoxically, therapeutic doses of oestrogens now no longer induce growth proliferation, but induce apoptosis. More recently, *ESR1* gene amplification was described in patient-derived mouse xenografts as a possible marker for hypersensitivity to oestradiol [28]. It would, therefore, be of interest in future studies to combine ^18^F-FES-PET/CT with analysis of *ESR1* gene amplification and mutation in tumour biopsies, in order potentially to improve the selection of patients for oestrogen therapy.

Although ER expression is required for response to endocrine agents, ER-positive tumours may still fail to respond, e.g., due to cross talk with other pathways. We, therefore, evaluated whether the addition of tumour and bone turnover markers could improve response prediction. The association of tumour marker response alone with the patient response classification was modest and did not add significantly to ^18^F-FES-PET/CT. For bone turnover markers, not the changes in bone markers during treatment, but the pre-treatment values were associated with time-to-progression. These markers are known to correlate with the number and size of bone metastases in breast and prostate cancer [15–17]. Therefore, high serum bone markers may be useful to identify patients that have a poor prognosis independent of the therapy given. Whether bone markers are of prognostic or predictive value needs to be addressed in larger studies, adhering to REMARK criteria [29].

We observed a clinical benefit rate of 37 % for oestradiol 6 mg orally daily, which is comparable to the study by Ellis et al. [4]. Our study is the second evaluating this low-dose regimen, as compared to the previous standard of 30 mg orally daily. The clinical benefit rate observed in our study in patients that were extensively pre-treated (median of four prior regimens) provides further evidence for the efficacy of oestradiol 6 mg daily. The 21 % of patients that terminated treatment prematurely due to toxicity was relatively high; grade 3 adverse events were noted in 42 % of the patients. The high incidence of toxicity, however, underlines the value of upfront predictive markers for this treatment.

## Conclusion

Patients with acquired endocrine-resistant metastatic breast cancer may paradoxically benefit from oestradiol therapy. The relatively low response rate and toxicity accompanying oestradiol therapy warrants exploration of potential biomarkers to predict response. ^18^F-FES-PET/CT may aid to identify patients that are unlikely to respond to oestradiol therapy.
